# Empirical Evaluation of the Possible Contribution of Group Practice of the Transcendental Meditation and TM-Sidhi Program to Reduction in Drug-Related Mortality

**DOI:** 10.3390/medicina59020195

**Published:** 2023-01-18

**Authors:** Michael C. Dillbeck, Kenneth L. Cavanaugh

**Affiliations:** Dr. Tony Nader Institute for Research on Consciousness and Its Applied Technologies, Maharishi International University, Fairfield, IA 52557, USA

**Keywords:** drug overdose death, social stress, consciousness, social experiment, Transcendental Meditation, TM-Sidhi program, interrupted time series analysis

## Abstract

*Background and Objectives:* CDC data indicate that the U.S. is experiencing a sustained epidemic of drug-related mortality, with such deaths exceeding a record 100,000 in 2021, up 47% from 2019. Opioids, especially the synthetic opioid fentanyl, account for approximately 75% of this mortality. This study evaluates a proposed Consciousness-Based^®^ approach that may possibly help reduce trends in drug-related fatalities by mitigating what WHO refers to as an “epidemic of stress” in society that helps fuel drug misuse and other negative public health trends. This approach involves providing support in public and private sector public health initiatives for individual and group practice of a subjective, evidence-based meditation procedure suitable for those of all educational, cultural, and religious backgrounds: the Transcendental Meditation^®^ (TM^®^) technique and its advanced aspect, the TM-Sidhi^®^ program. *Materials and Methods:* Segmented-trend regression analysis of monthly CDC data on U.S. drug-related fatality rates (*dfr*) from a prospective social experiment (2002–2016) was used to replicate and extend prior peer-reviewed research. *Results:* As hypothesized, (1) practice of the TM and TM-Sidhi program by a group of theoretically predicted size (√1% of the U.S. population) was associated with a statistically and practically significant reduction in *dfr* trend during the five-year “demonstration period” of the quasi-experiment; and (2) monthly *dfr* trend subsequently increased during the five-year follow-up period when the group fell below the required size (both *p*’s < 0.0001). The estimated total percent decrease in *dfr* during the demonstration period was 35.5%, calculated relative to the baseline mean. This decline was followed by total *dfr* increases of 11.8% and 47.4% relative to the demonstration-period mean during the two phases of the follow-up period. *Conclusion:* Existing evidence warrants implementation and further evaluation of this approach in U.S. public health initiatives.

## 1. Introduction

### 1.1. The Epidemic of U.S. Drug-Related Mortality

According to government statistics, the U.S. is experiencing a sustained epidemic of drug-related mortality, with U.S. drug-related fatalities reaching record highs in 2021. Since 1999, more than one million Americans have died from drug overdose, more than total U.S. battle deaths in all U.S. wars. Total drug-related fatalities were estimated at over 100,000 in 2021, more than double the total for gun-violence deaths and motor vehicle fatalities combined [1]. Annual drug-overdose mortality has been rising rapidly for years, but such deaths surged during the COVID-19 epidemic, rising 47% from 2019 to 2021 [2]. During the 12-month period ending in April 2021, opioids accounted for 75.4% of total drug-related deaths [3].

The dramatic rise in drug-related mortality has occurred despite a 44.4% reduction in opioid prescriptions between 2012 and 2021 [4]. Despite this reduction, illicitly manufactured fentanyl, fentanyl analogues, and stimulants (e.g., methamphetamine, cocaine) are killing Americans in record numbers. Currently, the main driver of drug overdose deaths is synthetic opioids (other than methadone) [5]. Because the synthetic opioid fentanyl is cheaper to produce and stronger than heroin, it is often mixed with heroin and other drugs. Fentanyl is up to 50 times more potent than heroin, contributing to the likelihood of unintentional overdose [1]. In 2020, 82.3% of opioid-involved overdose deaths involved synthetic opioids [5]. Annual overdose deaths have more than doubled since fentanyl began to be widely available in the U.S. [2]. Drug overdose mortality involving psychostimulants, such as methamphetamine, has also increased, with and without synthetic opioid involvement [3].

Drug-overdose fatalities cost the economy an estimated USD 1 trillion per year in health expenditures, reduced productivity, and other losses, according to a recent government report [1].

The devastation that opioids have caused to the U. S. population remains a source of embarrassment to the medical profession and pharmaceutical industry. Public health experts are searching for effective, evidence-based solutions to the epidemic of drug-related death, an epidemic unique to the U.S. among economically advanced nations; however, according to leading drug policy expert, Keith Humphreys at Stanford University, no one has a good answer for how to halt the spread of drugs such as fentanyl [2].

### 1.2. U.S. Epidemic of Stress

The use and effects of opioids is exacerbated by stress in society, which itself is a larger and more pervasive problem. Stress has been termed the “Health Epidemic of the 21st Century” by the World Health Organization (WHO) [6]. Recent polls conducted on behalf of the American Psychological Association (APA) suggest that Americans are experiencing an epidemic of chronic stress and burnout: U.S. stress levels have been pushed to alarming levels by a succession of events including a global pandemic, inflation, financial worries, a cascade of collective traumas from repeated mass shootings, and the war in Ukraine [7]. “Americans have been doing their best to persevere over these past two tumultuous years, but these data suggest that we’re now reaching unprecedented levels of stress that will challenge our ability to cope,” said Arthur C. Evans, Jr., APA’s chief executive officer [7]. Nearly one in four of the APA survey respondents reported they tried to cope with increased stress by increased drinking of alcohol. The survey did not ask respondents about increased use of opioids or other drugs. 

Likewise, work-related burnout and stress are at all-time highs across professions according to APA’s 2021 Work and Well-being Survey of 1501 U.S. adult workers [8]. According to the survey, 79% of employees reported experiencing work-related stress in the month prior to the survey. Negative impacts of work-related stress were reported by nearly 60% of employees: reduced motivation, lack of interest, or decreased energy and lack of effort at work. Other reported effects were cognitive weariness, emotional exhaustion, and physical fatigue. Work-related stress is estimated to cost U.S. businesses as much as USD 300 billion per year [6].

The WHO defines burnout as a syndrome resulting from unsuccessful management of workplace stress and characterized by feelings of energy depletion or exhaustion, increased mental distance from one’s job or negative feelings or cynicism related to one’s job, and reduced professional effectiveness [9]. 

An attempt to operationalize the idea of social stress was the work of Linsky et al. [10], who proceeded under the assumption that just as transitions in work that require adaptation are stressful independent of whether they are good or bad [11], transitions in other areas of life provide a measure of social stress.

Analyzing U.S. state data by quantifying a State Stress Index (SSI) to combine economic, family, and community stressors, Linsky and Straus [12] and Linsky et al. [10] found that the degree of social stress predicted the violent crime rate and also maladaptive behaviors. However, social stress so defined was less associated with mortality due to illness, illnesses whose morbidity at the individual level are exacerbated by stress. One of the possible reasons for this, noted by Linsky and Straus [12], is the unknown and variable time lag between serious morbidity and mortality, not to mention between serious stress and morbidity. This point is particularly salient for monthly time series data such as the data of the present research. The public health indicator analyzed in this study, the drug-related fatality rate (*dfr*), is one where mortality is directly influenced by stress, rather than one where the disease state develops over time and whose time course may be inconsistent and hard to specify.

### 1.3. Structure of the Paper

The purpose of this study is to evaluate a proposed Consciousness-Based approach to possibly help reduce drug-related fatalities by preventing and mitigating the stress on the societal level that helps to fuel drug misuse and other negative public health related trends.

The remainder of this paper is structured as follows. In Section 2, we discuss the proposed Consciousness-Based approach: group practice of the TM and TM-Sidhi program. This approach was suggested by the findings of previous quasi-experimental, longitudinal research [13] that found strong empirical support for the hypothesis that practice of TM and its advanced procedure, the TM-Sidhi program, by a group of theoretically predicted size was associated with statistically and practically significant reduction of societal stress, as indicated by a decrease in rising monthly baseline trends of U.S. drug-related fatality rates. The group was located at Maharishi International University (MIU) in Fairfield, Iowa USA (hereafter referred to as the “MIU group” or the “TM-Sidhi group”). The empirical aspect of the current study seeks to replicate and extend this previous research on *dfr* trends using an expanded data sample that includes a five-year follow-up period and longer baseline and quasi-experimental “demonstration” (or “treatment”) periods.

Section 3 reviews previous peer-reviewed empirical research that examined the hypothesized societal impacts of the group practice of the TM and TM-Sidhi program on other public health related indicators. Section 4 describes the data and data sources of the current study, discusses the quasi-experimental research design, and formulates the segmented-trend regression model used for the interrupted time series regression analysis. Section 5 operationalizes the research hypotheses in terms of the estimated coefficients of the regression model, discusses the results of the hypotheses tests, and reviews the battery of diagnostic tests used to assess statistical conclusion validity. Section 6 summarizes the empirical findings and considers alternative possible explanations of the empirical results. Section 7 presents the conclusions.

## 2. Consciousness-Based Approach to Decreasing Drug-Related Mortality

### 2.1. Transcendental Meditation Technique

The Transcendental Meditation (TM) technique is described as a Consciousness-Based approach to reducing and preventing stress on the individual level, mitigating the adverse effects of stress, enhancing health, and promoting unfoldment of latent potentialities of human consciousness. TM was introduced to the West from India in 1958 by Maharishi Mahesh Yogi, the noted scholar of the ancient Vedic tradition of knowledge and scientist of consciousness who encouraged scientific research on the physiological, psychological, and sociological effects of TM practice. This evidence-based meditation procedure is said to be suitable for those of all educational, cultural, and religious backgrounds. The TM technique is practiced for 20 min twice daily sitting comfortably with eyes closed [14]. It is described as a systematic procedure for allowing mental activity to spontaneously settle down and thereby open awareness to conscious experience of “pure consciousness,” in which awareness is awake to itself alone, without thought or perception [14,15].

It is important to note that there are many different meditation practices and that it is possible to characterize meditation techniques by differing purposes, different procedures, and different patterns of EEG and other measures of brain activity [16]. In the “Open Monitoring” category of meditation practices (e.g., Zazen and mindfulness), practitioners seek to simply be aware of the moment-by-moment flow of subjective experiences. The “Focused Attention” category of meditation practices includes techniques that try to control the mind, for example, to keep it focused on bodily sensations (Zen), breathing (Vipassana), or the feeling of loving kindness or pure compassion (loving kindness or compassion meditation). Transcendental Meditation is in the “Automatic Self Transcending” category, effortlessly transcending the steps of meditation practice to experience transcendental consciousness, pure consciousness, pure inner wakefulness.

We are using Transcendental Meditation in this study because it is a uniform practice and because its social effects have been the subject of prior research (see Section 2.6 and Section 3).

More than 400 peer-reviewed research papers reporting an extensive range of physiological, psychological, and sociological effects of TM practice have been published. Eight volumes of collected research papers on TM and the TM-Sidhi program have been published to date, (e.g., [17]); these articles are indexed in an online database at https://researchtm.net/.

Published research supports the conclusion that the increased inner wakefulness during TM corresponds to a unique psychophysiological state of reduced stress and restful alertness [18]. Alertness is indicated by increased coherence in the electroencephalogram (EEG) measured during TM practice. This state of restful alertness or reduced physiological stress is also evident in participants outside the practice period (lower respiration rate, lower heart rate, lower plasma lactate, fewer spontaneous skin resistance responses) [19].

The state of restful alertness experienced during TM practice is indicated by higher alpha frontal EEG coherence [20], subsequently narrowed to alpha 1 (8–10 cps) frontal coherence [16,21], and broadband frontal coherence both inside and outside of meditation [22]. Magnetoencephalographic research suggests that the source of the EEG alpha is the medial prefrontal cortex and anterior cingulate cortex [23]. Increased integration of brain functioning is also found longitudinally in those practicing TM [20,21,24].

Other physiological research, including reduced cortisol during the practice and more effective cortisol response to stress, indicates that the physiological changes during TM practice are counter to those found during stress and these changes support recovery from stress [25,26,27].

### 2.2. Research on Health-Related Behavioral Effects of TM Practice

Research on longitudinal effects of TM practice that indicate reduction of stress-related conditions include reduced symptoms of PTSD and depression [28], decreased anxiety [29], decreased hostility and aggression in prison inmates [30], reduced recidivism by convicted felons [31], reduced compassion fatigue and burnout among nurses [32], and reduced burnout, perceived stress, depression, and fatigue in teachers [33]. Other extensive TM research on psychological variables can be considered in the context of theories of human development, (e.g., [34,35]).

There is also a large body of research on the stress-reducing effects of this procedure for physiological health. This state of restful alertness during Transcendental Meditation has been found by meta-analysis to decrease stress-related conditions such as high blood pressure [36,37,38] as well as reduce cardiovascular problems in patients diagnosed with coronary heart disease, including risk for mortality, myocardial infarction, and stroke [39]. See Schneider and Carr [40] for a review of effects of TM practice on cardiovascular health.

The TM program has been shown to be effective in the treatment of substance use disorder (see Hawkins [41] for a review). Meta-analysis indicates that application of this approach over time leads to decreases in substance misuse that do not result in relapse [42]. This meta-analysis of 198 studies also reported that the TM program was highly effective relative to other standard approaches for reducing drug abuse, with 0.83 average effect size for the TM program as compared to 0.47 for peer-influence approaches, and 0.13 for prevention-oriented education programs. Effect sizes for TM practice were even higher for the strongest research designs (0.91) and for heavy users (1.16).

In a theoretical review paper, Walton and Levitsky [43] provide a neuroendocrine model for the effects of chronic stress and its contribution to drug addiction. In light of this model, the authors review empirical research on the physiological effects of Transcendental Meditation, and propose mechanisms whereby TM creates increased physiological balance, thus promoting reduction in addictive behaviors.

### 2.3. Reduction of Societal Stress and Its Effects

The principal empirical focus of the current study is evaluation of the reduction of drug-related fatalities by reducing stress in the collective consciousness of society through group practice of the TM and its advanced aspect, the TM-Sidhi program. The theoretical perspective informing the current research is discussed in greater detail in previously published research (e.g., [44]).

As early as 1962, Maharishi predicted that measurable reductions of social stress and resulting negative societal trends would begin to be measurable when approximately 1% of the population of a nation, province, or city practice the TM technique individually [45].

In 1976, Maharishi introduced the advanced TM-Sidhi program, the purpose of which is described as accelerating the integration of the inner experience of pure consciousness during TM with daily activity outside of meditation [46]. In discussions with physical scientists in the light of ancient Vedic knowledge, he formalized the prediction that when a group practices the TM-Sidhi program together, only the square root of one percent of the population (√1%)—as compared with 1% practicing Transcendental Meditation individually—would be required to create a calming and orderly influence in society [46,47]. The square root term is found in coherent phenomena in physical systems, in which the combined intensity of coherent elements, such as constructive inference of light waves in a laser, can be proportional to their square [48].

Due to the nonlinear nature of the √1% formulation, the prediction is that it would be possible to influence very large populations: for example, the square root of one percent of a large city of 9 million is 300 individuals, and for the current U.S. population it is slightly less than 1800. As a consequence, it is feasible to do quasi-experimental studies [49] by bringing a group together either on a temporary or stable basis and measuring changes in social parameters on a fine time scale.

The collective effects on society have been predicted by Maharishi Mahesh Yogi to occur independent of any attempt to think about society during the practice of TM or the TM-Sidhi program, but rather as a spontaneous byproduct of individuals stimulating the more universal level of their own awareness [46].

In this paper, we use the term “Maharishi Effect” to refer to the predicted effect on social indicators of either 1% of individuals in society practicing TM individually or the √1% of the population practicing the TM and TM-Sidhi programs together in a single group. Published research on the Maharishi Effect hypothesis has now been replicated at the city, state, and national levels.

### 2.4. Principles from Ancient and Contemporary Theorists

Over a period of five decades, Maharishi Mahesh Yogi (1917–2008) revived and refined procedures for development of consciousness from the ancient Vedic tradition of knowledge of consciousness preserved in India. He also encouraged scientific research on application of these procedures to diverse areas of life, (e.g., [17]). This comprehensive revival and reinterpretation of the Vedic knowledge of consciousness has been termed “Maharishi’s Vedic Science and Technology^®^ (MVST^®^)”.

Unlike most contemporary Western theories of mind and consciousness, MVST posits the existence of a nonlocalized, interpersonal dimension of consciousness that underlies the more expressed, localized values of individual consciousness as well as the collective consciousness of society [44]. Recently, Harvard and MIT-trained neuroscientist Dr. Tony Nader, MD PhD, who worked closely with Maharishi for over 20 years, has formulated the fundamental principles expressed in MVST in a framework of formal logic [47,48]. See Cavanaugh, Dillbeck and Orme-Johnson [44] for further discussion of the relation of MVST to other current and historical theories of mind, consciousness, and the concept of collective consciousness.

As brought to light by Maharishi from the ancient Vedic knowledge of consciousness and further elaborated by Nader, consciousness is viewed not as an emergent property of matter generated by functioning of the brain and human nervous system, but rather as a fundamental reality at the basis of all matter and diversity. Pure consciousness is described as the essential nature of life—a unified, unbounded, field of pure intelligence, beyond the limitations of space and time [14,47,50].

This fundamental, unified field of nature’s intelligence is described as the transcendental source of all natural laws governing the evolution of life on earth and the entire universe [46,47,50]. The fundamental principle of this Vedic understanding of consciousness is that “consciousness is all that there is” [47,50,51].

Noted quantum physicist John Hagelin has pointed out the striking similarities between this Vedic view of consciousness as a universal field that is the transcendental source of all natural laws and the concept of the universal quantum field, the unified field, described in contemporary unified field theories in physics [48]. As discussed in greater detail in Cavanaugh et al. [44], the extra conceptual step in MVST is that this universal field described by quantum physics has the property of consciousness; it is an unbounded field of consciousness.

Maharishi held that practice of the TM technique allows individual awareness to effortlessly open to this most fundamental level of consciousness—pure consciousness or pure awareness [14,15]. The field of pure consciousness is said to underly the waking, dreaming, and sleeping states of consciousness. Pure awareness is also viewed as underlying the collective consciousness of society, which is defined as the wholeness of consciousness of the individuals comprising the social group: family, city, province, or nation, e.g., “group consciousness” [44,52].

Maharishi [51] (p. 59) explains that a positive, life-supporting influence is enlivened in society when individuals transcend the subtlest level of thought and consciously contact the field of pure consciousness:

“Because consciousness is the basis of all that is there—here, there, and everywhere—it is the quantum level of life, the very basic level of life. If the attention reaches that level, what happens is like the small pebble falling on the silent bed of the water. A small pebble falls, creating impulses. Those impulses reach all the far places and all the water. Just like that, when the conscious mind of one single individual transcends, we can imagine the thrills being created on that silent level of consciousness, which is the omnipresent reality. This pulsating consciousness of the individual creates impulses of life all over, and because this is the very fundamental level of everyone, everyone’s thinking, everyone’s consciousness is influenced by that. It is very easy to understand.”

Maharishi [51] (p. 59) further explains that when even as few as approximately 1% of individuals in society practice TM, this enlivens the field of pure consciousness that underlies the collective consciousness of society, positively influencing societal trends: “The whole society becomes more positive in its trends, more positive in its thinking. The awareness of the whole population is influenced tremendously. That is why the criminals change, negativity changes.” The same enlivenment of pure consciousness in society generated by 1% of the individuals in society practicing TM is said to be produced when the √1% of the population practice the TM and TM-Sidhi programs together in a single group [46]. Maharishi [46] (pp. 163–164) describes that the positive transformations in society are generated from the level of the universal field of consciousness: “The effect produced [on society] is from the level of the unified field. Because the unified field is the unmanifest basis of the whole creation, the influence spreads throughout the world. It’s just like the effect when you water the root and the nourishment reaches every leaf, branch, flower, and fruit.”

From the viewpoint of the understanding of consciousness brought to light from the Vedic tradition of knowledge by Maharishi, just as an individual’s behavior depends on the quality of their individual consciousness, the quality of behavior in society is said to depend on the quality of collective consciousness of society [52]. The quality of collective consciousness is viewed as the primary determinant of the quality of life in society and of social change [46,52].

Maharishi further posits that there is a reciprocal relationship between individual and collective consciousness [52]. Thus, changes in stress and tension in individual consciousness will influence the quality of collective consciousness, and vice versa [53,54].

The consequence of the accumulation of stress in individual and collective consciousness is said to be that societal problems such as drug abuse, accidents, illness, violence, conflict, crime, and other non-life-supporting, negative trends become more prevalent: “If the collective consciousness of the country is under stress, then incoherent and conflicting tendencies will predominate in society and problems, turbulence, and violence will characterize the nation” [55] (p. 8).

### 2.5. The Concept of Collective Consciousness: Historical Precedents and Contemporary Examples

As discussed in Cavanaugh et al. [44], historical precedents from Western thought for the concept of collective consciousness or group mind include, among others, the concept of “conscience collective” discussed by French social thinker Emile Durkheim [56,57]; the concept of “group mind” or “national mind” considered by William McDougall [58], one of the founders of social psychology; and the hypothesized “continuity of consciousness” underlying the experience of ordinary waking consciousness as discussed by Gustav Fechner, one of the founders of experimental psychology [59]. Contemporary examples include more than 20 years of research at the Global Consciousness Project at Princeton University’s Engineering Anomalies Research Lab that has reported empirical evidence said to support the existence of global consciousness [60]. Additionally, a series of empirical studies in behavioral finance has recently investigated the relationship between financial market performance and measures of national mood (e.g., [61]). Common expressions referring to the concept of a collective aspect of consciousness, or group mind, include “team spirit” in sports, “employee morale” in business, “national mood” or “public opinion” in public opinion polling, “investor sentiment” or “investor mood” in finance, and “army morale” in the military.

### 2.6. Prior Empirical Research on the Current Social Experiment

The present study extends previous research on the same prospective quasi-experiment. This earlier research assessed impacts of group practice of the TM and TM-Sidhi program by the MIU group on diverse public health related indicators—including rates of drug-related fatalities—using a 2002–2010 subset of the expanded monthly data set (2002–2016) analyzed in the current study. Using segmented-trend (spline) regression to analyze baseline and demonstration-period data through 2010, these studies reported decreased trends for each of the following fatality rates relative to baseline trend. For each fatality rate we give the *t*-statistic, *p*-value, and standardized effect size (ES) Cohen’s *f* for regression coefficients [62]: decreased national drug-related death, *t*(87) = −4.16, *p* < 0.001, *f* = −0.449 (30.4% reduction) and decreased national infant mortality, *t*(86) = −4.50, *p* < 0.001, *f* = −0.482 (12.5% reduction) [13]; decreased national homicide, *t*(84) = −10.17, *p* < 0.001, *f* = −1.11 (21.2% reduction) and decreased urban violent crime, *t*(83) = −6.14, *p* < 0.001, *f* = −0.674 (18.5% reduction) [63]; decreased national traffic fatalities, *t*(87) = −8.55, *p* < 0.001, *f* = −0.917 (20.6% reduction) and decreased national fatalities due to other accidents, *t*(86) = −3.82, *p* < 0.001, *f* = −0.412 (13.5% reduction) [64]; and decreased urban murder, *t*(84) = −8.89, *p* < 0.001, *f* = −0.970 (28.4% reduction) [65]. The percent reductions reported in parentheses are raw effect sizes given by the total predicted reduction for each fatality rate during the demonstration period divided by its baseline mean and multiplied by 100.

Standardized ES were calculated from data reported in the original papers, with Cohen’s *f* given by the *t*-ratio (or square root of the *F* statistic) for a variable’s coefficient in the regression equation divided by the square root of the residual degrees of freedom [62,66]. In this metric, 0.59, 0.39, and 0.14 are considered large, medium, and small effects, respectively [62]. The unsquared ES metric is reported, rather than *f* ^2^ because unsquared measures are said to better indicate the relative magnitude of effects across variables [67]. For details, see file S1_Appendix_A in the online Supplemental Files for this article at the Open Science Framework (OSF) repository (https://osf.io/vbkfc/, accessed on 15 January 2023).

A recent study [44] further extends and updates the analysis of monthly CDC data on U.S. homicide rates during the social experiment examined in the present study. Segmented-trend regression analysis was used to analyze an expanded data sample that includes an extra year (2011) of demonstration-period data. Replicating the finding of Dillbeck and Cavanaugh [63], the updated study found support for the hypothesis of a reduced trend slope for monthly homicide rates relative to baseline trend during the demonstration period (*t*(163) = −6.08, *p* < 0.0001, *f* = −0.476, a medium-large effect). Practical significance was indicated by the 19.3% reduction in homicide rate, as measured by the total predicted reduction in the rate during the demonstration period relative to baseline trend divided by the mean baseline rate. As discussed in Section 4.2, the total predicted reduction in homicide rate relative to the baseline trend is a standard measure of “treatment effect” in interrupted time series (ITS) analysis of quasi-experiments [68,69].

Cavanaugh et al. [44] also extend the prior findings of Dillbeck et al. [63] by analyzing monthly U.S. homicide rates during a five-year follow-up period (January 2012 to December 2016) when the size of the TM-Sidhi group at MIU fell below the required √1% level. Analysis of data from this “ABA” quasi-experimental ITS design offered strong empirical support for the hypothesis of an increase in trend for the homicide rate during the follow-up period relative to the demonstration-period trend. This hypothesis was supported for both subperiods of the five-year post-demonstration period: a statistically and practically significant increase in homicide trend (2012–2014) (*t*(163) = 4.10, *p* < 0.0001, *f* = 0.321, a medium effect) and an increased trend 2015–2016 (*t*(163) = 10.06, *p* < 0.0001, *f* = 0.788, a large effect). The monthly homicide rate increased by a total of 11.8% in 2012–2014 and by 28.5% in 2015–2016, respectively, relative to the mean rate for the demonstration period.

A second recent study analyzes 17 years of annual data from the same social experiment evaluated in the current study [70]. Segmented trend regression analysis of time series with ARIMA modelling of regression errors found statistically and practically significant reductions in trend compared to baseline trends 2000–2006 in eight rates of fatalities and violent crime, as well as a composite index of these measures, during the same years as the monthly demonstration period of the current *dfr* study, 2007–2011. During the demonstration period when the TM-Sidhi group reached or exceeded the predicted required threshold of √1% of the U.S. population (1725), there were significant and meaningful trend reductions in indicators of “national stress”: rates of homicides, rape, aggravated assault, robbery, infant mortality, drug-related deaths, motor vehicle fatalities, fatalities due to injuries in youths ages 10–19, and in the composite “national stress index” of the eight variables (index *p* < 0.0001).

Moreover, from 2012 to 2016, when the size of the MIU group decreased to below the required √1% threshold, trends for all stress indicators increased significantly. Standardized effect sizes and percentage changes in all variables, as well as substantial estimated reductions in fatalities and the number of crimes, supported practical significance of the findings (for details, refer to [70]). The reported findings for annual data on rates of homicide and drug-related death replicate those for monthly data reported in Cavanaugh et al. [44] and the current study, respectively.

## 3. Other Previous Empirical Research on the Maharishi Effect

### 3.1. Research on Public Health Related Indicators: City Level

At the city level, two studies [71,72] have reported reductions in violent crime in Washington, D.C. (in the case of the former, study of all FBI violent crimes (*t*(100) = −2.52, *p* < 0.05, with effect size *f* = −0.252); the latter study looked at, in particular, violent crimes against persons (homicide, rapes, and assaults) (*t*(39) = −5.47, *p* < 0.0001, *f* = −0.876) during periods when there were large groups of participants in the TM-Sidhi program established in the metropolitan area. Both studies used Box-Jenkins transfer function (TF) analysis, as the numbers were more than the predicted threshold. In almost all cases, the parameter indicating the effect of the large group of TM-Sidhi program participants was one-tailed, since the direction of effect is predicted by theory, while for other parameters a two-tailed test is more appropriate, since the direction of effect is not predicted.

In the UK, a TM-Sidhi group not far from Merseyside grew to exceed the predicted size to influence the Merseyside metropolitan area in March 1988. Time series (TS) analysis of data from 1979 to 1991 indicated a significant effect of reduced crime rate by 13.4%, (*t*(141) = −4.68, *p* < 0.0001, *f* = −0.394) beginning in March 1988, a crime drop independent of economic trends and unique in the nation at that time [73].

In a prospective research project in 1983 in Israel, a temporary group of TM-Sidhi program participants was brought together in Jerusalem for two months [74]. The group varied in size throughout this period, sometimes over the required number for Jerusalem, or for Israel as a whole, or for Israel and Lebanon together (Israel had troops within part of Lebanon at that time). Collecting multiple variables that were available daily either for Jerusalem or Israel as a whole, the authors found significant improvements in equally weighted indices of quality of life at the city and national levels (for the city, *t*(55) = 2.85, *p* < 0.01, *f* = 0.384; for the nation, *t*(54) = 4.00, *p* = 0.0001, *f* = 0.544). Improvements were also found for measures of war intensity (*t*(55) = –2.71, *p* < 0.005, *f* = −0.365) and war deaths (*t*(55) = −2.12, *p* < 0.02, *f* = −0.286) from the Lebanese conflict derived by content analysis of daily news sources. These findings were evident in both TS impact-assessment analysis for the relevant demonstration periods as well as time series TF methods [75,76]. Time series cross-correlation and TF analyses indicated that the participant numbers had a leading effect in time on the dependent variables, rather than the other way around. In response to critiques suggesting possible confounding factors [77,78]; the authors published several re-analyses of the data, which supported their initial findings [79,80].

### 3.2. Research on Public Health Related Indicators: Province or State Level

At the state level, in 1978 groups of TM-Sidhi program participants were organized in Rhode Island for three months, sufficient in size to have a predicted influence on the state as a whole. TS impact analysis [76,81] of monthly data indicated improved overall quality of life in Rhode Island during the three-month period compared to that in the similar state of Delaware on an equally weighted index of eight behavioral variables (*t*(68) = 2.64, *p* < 0.01, *f* = 0.320). The index included crime rate, motor vehicle fatality rate, and other social indicators [82].

Similar significant reduction of crime rates were reported in TS impact-assessment studies at the state level in which large groups of TM-Sidhi program participants came together temporarily on courses [82]: for the Union Territory of Delhi, India (the national capital territory), a reduction of daily Indian Penal Code totals (*t*(260) = −5.12, *p* < 0.0001, *f* = −0.318); for Metro Manila (the national capital territory), a reduction of weekly crime index totals, equivalent to the FBI Uniform Crime Index in the U.S. (*t*(91) = −2.83, *p* < 0.005, *f* = −0.298); and for Puerto Rico, a reduction of monthly Type 1 crimes, comparable to the FBI Uniform Crime Index (*t*(160) = −2.02, *p* < 0.025, *f* = −0.160).

### 3.3. Research on Public Health Related Indicators: National Level

Additionally, at the national level in the U.S., the group of TM-Sidhi program participants at MIU reached the required number of √1% in the early 1980s. Dillbeck [83], using TS methods, found a significant reduction in fatalities due to homicide, suicide, and motor vehicle fatalities during 1982–1985 when the size of the group reached √1% of the population (*t*(136) = −2.47, *p* < 0.01, *f* = −0.212); and TF results indicated that although the size of the group led changes in fatalities, the reverse was not true. 

Davies and Alexander [84] extended prior research by Orme-Johnson et al. [74] on Israel to examine the conflict in Lebanon more extensively, looking at all seven occasions over a 2.25-year period during 1983–1985 when there were temporary groups of TM-Sidhi program participants either in Lebanon, near to it (including group TM-Sidhi practice in Israel), or further away that were large enough to have a predicted effect on the ongoing conflict there according to the √1% principle. Using a daily database created from nine international and regional news sources by an independent Lebanese rater blind to these hypotheses, TS impact analysis indicated reduced conflict intensity (*t*(810) = −5.81, *p* < 0.0001, *f* = −0.204), decreased conflict fatalities (*t*(814) = −6.45, *p* < 0.0001, *f* = −0.226), and increased cooperation by factions (*t*(810) = 4.96, *p* < 0.0001, *f* = 0.174) during the seven assemblies of TM-Sidhi program participants in contrast to all other days. The analysis controlled for seasonality and trends in the data, temperature, holidays, or weekends.

An overview of additional empirical research on the Maharishi Effect at the national level is given in Section 2.6, which describes previous studies on U.S. monthly drug-related death and other public health related indicators associated with the prospective social experiment examined in the current research.

## 4. Methods

### 4.1. Data Definitions and Sources

Monthly drug-related fatality totals for January 2002 through December 2016 were obtained from the WONDER online database of the National Center for Health Statistics of the U.S. Centers for Disease Control and Prevention (CDC) [85]. Data are from the Multiple Cause of Death Files, 1999–2016 (ICD-10 codes W00-X59 “Other external causes of accidental injury”). The CDC data includes mortality from any type of drug and any circumstances of death provided that the death certificate specifies that drugs are the essential cause of death. Drug-related fatality totals were then converted to monthly fatality rates per one million population (*dfr*) using not-seasonally adjusted monthly U.S. population estimates from the U.S. Bureau of Economic Analysis (series POPTHM) obtained from the FRED database at the Federal Reserve Bank of St. Louis [86]. The monthly fatality rate *dfr* is given by the monthly fatality total divided by the monthly U.S. population estimate in millions. The daily number of group participants in the afternoon meditation session at MIU (provided by the MIU Department of Development of Consciousness) was converted into monthly averages. The data used in the regression analyses are open source but also are posted online with other Supplemental Files at the OSF repository for this study: https://osf.io/vbkfc/.

### 4.2. Interrupted Time Series (ITS) Research Design

An ITS study design is used to empirically test a hypothesized reduction in the regression predicted value of *dfr* relative to the baseline trend during the demonstration phase of the quasi-experiment. We also test the hypothesis of an increase in predicted value of *dfr*, relative to the demonstration-period trend, during the follow-up phase after the “treatment” phase ends. Thus, the current study employs an “ABA,” or “baseline reversal,” quasi-experimental ITS design [87]. The study design is termed ITS because it is expected that introduction of the program or quasi-experimental “treatment” being evaluated will “interrupt” or alter the trend and/or level of the time series after its introduction.

When randomized, controlled experiments are not feasible, ITS designs are appropriate for the analysis of the longitudinal impacts of new programs, policy changes, laws, or other events [49,88,89]. Such prospective quasi-experimental designs are inherently stronger than ex-post multiple regression analyses of archival data on multiple correlated variables [89].

ITS analysis is popular in the program evaluation literature [68,69] and has been widely applied in many other areas of the social and other sciences (e.g., [76,81]). Even in the absence of a comparison group, ITS designs are considered to be appropriate for causal inferences in quasi-experiments because: (1) their control over regression to the mean [68,87] and (2) because the TS behavior of the outcome variable during a baseline period prior to introduction of the new event or program can be used to estimate an empirically based “counterfactual” TS for calculation of treatment effects [49,68,89]. Projection of the baseline trend into the demonstration period provides a counterfactual TS for comparison with the observed *dfr* series. A standard measure of “treatment effect” (TE) in single-group quasi-experimental designs is the difference between the predicted value of the outcome variable at the end of the demonstration period and the corresponding predicted value for the counterfactual TS [68,69,90].

Historically, Box-Tiao impact-assessment analysis [76,81] has been the most frequently used statistical approach in ITS analysis. However, when outcome variables such as *dfr* exhibit nonlinear behavior and when research questions focus on impacts on trends (possibly with accompanying level shifts), segmented-trend regression, including the special case of linear spline analysis [91], can be a useful approach for estimating program effects in quasi-experiments [68,90,92]. By contrast, ITS modeling using Box-Tiao impact-assessment and Box-Jenkins transfer function methods [75,76,81,93] often involves differencing of the dependent variable, thus removing any trends in the data.

### 4.3. Prospective Social Experiment

Like previous research on this social experiment, the baseline, demonstration, and follow-up periods were defined in terms of the mean monthly number of participants in the afternoon session of the group practice of the TM and TM-Sidhi program at MIU. The MIU group is composed of students, faculty, staff, and other community members who gather to practice these technologies of consciousness together twice daily, before and after the school or work day.

**Figure 1:**
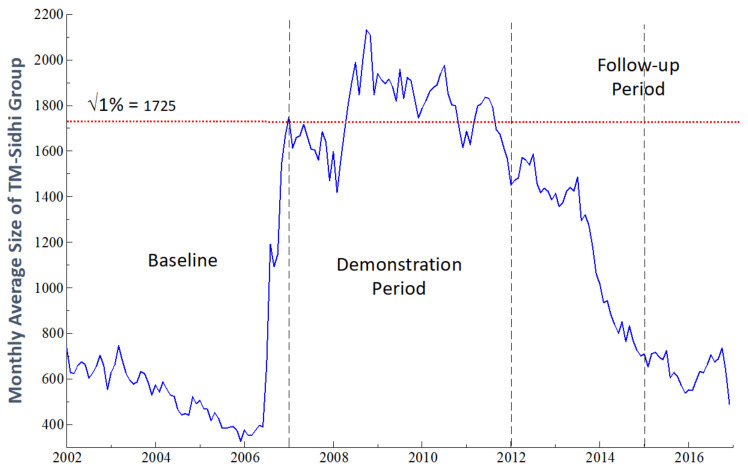
Monthly mean number of individuals practicing Transcendental Meditation and the advanced TM-Sidhi program together in a group at Maharishi International University, Fairfield, Iowa, 2002–2016.

As shown in Figure 1, as of mid-summer 2006, the size of the MIU group had displayed a declining trend for more than three years. In July 2006, MIU launched an initiative to increase the average group size from less than 400 in June 2006 to approximately the √1% of the U.S. population—1725 group participants, based on the U.S. population of 297 million at that time. Practitioners of the TM and TM-Sidhi program in the U.S. and abroad were invited to participate in a special program to expand the group size. To further increase the number of participants, visiting TM and TM-Sidhi program experts from India joined the group starting in October 2006. Supporting grants for increasing the size of the group were received by MIU from the Howard and Alice Settle Foundation.

Prior to the beginning of the quasi-experiment (and in its early weeks), press releases and other publicity predicted that reductions in rates of fatalities due to accidents, illness, homicide, violent crime, and other negative societal trends would be a measurable impact of the MIU group when its size reached approximately the √1% of the U.S. population. These predictions were based on previously published empirical research and theoretical principles.

### 4.4. Baseline, Demonstration, and Follow-Up Periods

The period January 2002 to December 2006 serves as the baseline period for the study. For the MIU group, the mean daily number of participants for the month first reached the predicted, approximate critical threshold in January 2007 and remained above or near threshold through 2011 (see horizontal dotted line in Figure 1). During 2007, the size of the group continued to be above or near this level (averaging 1636, or 95% of the critical threshold). Although the MIU group size was somewhat below threshold for the initial four months of 2008, the average monthly size of the group for the year was 1824.

The annual mean monthly size of the group was 1815 (105% of threshold) for the five years January 2007 through December 2011. Therefore, we define the five years 2007–2011 as the demonstration period, or *phase2*, of the study (the period between the two vertical dashed lines in Figure 1). During this period, the average monthly size of the MIU group exceeded approximately the √1% of the U.S. population, and for each of the five years, averaged at least 95% of the approximate √1% threshold.

December 2011 is defined as the end point of the demonstration period. In that month the number of TM-Sidhi group participants fell from above threshold to 91% (1566) of the approximate critical threshold of 1725. Therefore, the months of January 2012 through December 2016 comprise the follow-up phase of the study. During this period, group participation declined substantially due to reduced funding. In 2012, average monthly group participation declined to 1448, fell further to 1338 in 2013, and then declined sharply to 837 in 2014, falling further to 654 in 2015, and 628 in 2016.

The baseline period is referred to as *phase1* of the social experiment, while the demonstration period is denoted as *phase2*. The follow-up period is divided into two subperiods, January 2012–December 2014 (*phase3*) and January 2015–December 2016 (*phase4*), respectively; as discussed in Section 4.7 this empirically based subdivision was necessary to produce satisfactory diagnostic tests for the regression model.

### 4.5. Testable Hypotheses Examined in the Current Study

The purpose of the empirical aspect of the current paper is to replicate and extend the previous study of drug-related fatality trends 2002–2010 described in Section 2.6 [13]. The current empirical analysis employs segmented-trend regression modeling, a form of interrupted time series analysis, to test the two research hypotheses described below.

The quasi-experimental design of the current study incorporates a five-year baseline period and extends prior research by adding a five-year follow-up period to empirically test a hypothesis that predicts an increase in *dfr* trends after the end of the demonstration phase of the study. We also expand the demonstration period of the previous study from four to five years. During the demonstration period, the number of participants practicing the TM and TM-Sidhi program together in a group located at MIU was above or near the square root of 1% of the U.S. population at that time—the theoretically predicted, approximate size sufficient to predict positive measurable effects on trends in drug-related fatality rates and other indicators of public health and quality of life.

We test two research hypotheses. Hypothesis 1 states that during the demonstration period of the social experiment (January 2007 to December 2011) there would be a decrease in the regression predicted value (or fitted value) of the monthly drug-related fatality rate relative to that predicted by continuation of the five-year baseline trend (January 2002 to December 2006).

As mentioned in Section 4.2, a standard measure of ITS “treatment effect” (TE) is given by the difference between the regression predicted value of *dfr* at the at the end of the ITS demonstration period and the corresponding predicted value for *dfr* based on counterfactual projection of the baseline trend through the end of the period. In the current study, the TE for the demonstration period (TE_1_) is calculated by subtracting the counterfactual predicted value of *dfr* at the end of the demonstration period from the corresponding predicted (or fitted) value of *dfr* based on the full regression model. Thus, equivalently, Hypothesis 1 can be stated more concisely as predicting that the TE for the demonstration period should have negative sign (TE_1_ < 0).

Hypothesis 2 states that there would be an increase in the regression predicted value of *dfr* relative to the counterfactual continuation of the demonstration-period trend during the two follow-up subperiods of the study when the number of participants in the MIU group fell below the theoretically predicted critical threshold of approximately the √1% of the U.S. population. Treatment effects for the two trend segments of the follow-up period (TE_2_ and TE_3_) of the study are defined analogously to TE_1_. Thus, Hypothesis 2 states that TE_2_ and TE_3_ should have positive sign.

Note that the TE for any trend segment of the segmented-trend regression model will reflect the combined effect of any change in both *dfr* trend slope and the starting value for the segment trend (intercept or level).

In Section 5.4, we operationalize these two research hypotheses in terms of the estimated “long-run multiplier coefficients” of the segmented-trend regression model for *dfr* described in Section 4.7.

### 4.6. Time Series Plots of Data

**Figure 2:**
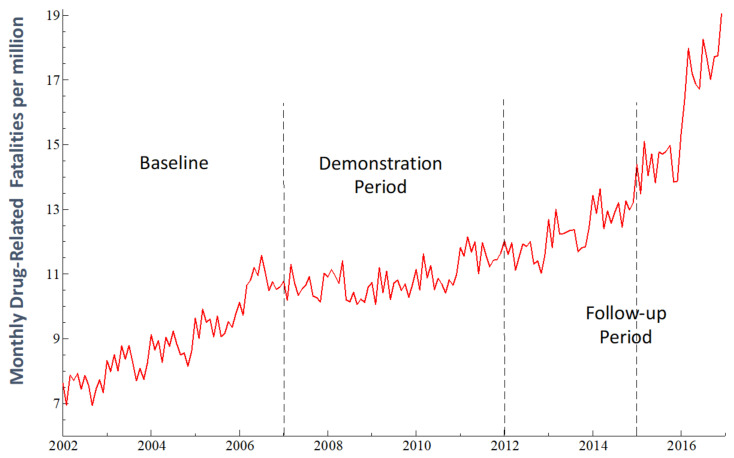
Monthly U.S. drug-related fatality rate per million population 2002–2016.

The general pattern of TS behavior for *dfr* shown in Figure 2 for January 2002 to December 2016 (sample size *N* = 180) appears to be broadly consistent with Hypotheses 1 and 2. A steeply rising *dfr* baseline trend with apparent seasonal variation around the trend is followed by an apparent downward level shift plus a reduction (flattening) in trend slope. Consistent with Hypothesis 1, these changes imply a cumulative total reduction in *dfr* during *phase2* relative to that predicted by continuation of the *phase1* trend.

Consistent with Hypothesis 2, the *dfr* trend slope increases during *phase3* (January 2012–December 2014) relative to the *phase2* trend with no apparent level shift, thus implying a cumulative increase in the predicted value for *dfr* relative to that which would be predicted by continuation of the *phase2* downward trend.

During *phase4* (January 2015–December 2016), the further increase in the rising *phase3 dfr* trend (with no level shift) is associated with an accelerated decline in the size of the MIU group to levels similar to those just prior to the mid-July 2006 onset of the social experiment. This *phase4* increase in *dfr* is also consistent with Hypothesis 2.

In social science, the precise timing and form of dynamic effects in ITS studies are almost always empirically determined (e.g., see [76,81]). However, the slower increase in *dfr* trend during *phase3* followed by an accelerated rise during *phase4* appears to be consistent with previous research [72] suggesting that the impact of such a TM-Sidhi group may be expected to decline relatively slowly when the group declines in size after exceeding the critical threshold for an extended period. When the group size falls below the √1% threshold, theoretical considerations suggest that the level of societal stress in collective consciousness may be expected to begin building up again, gradually eroding the gains of the demonstration period.

### 4.7. Segmented-Trend Regression Model

To evaluate the research hypotheses of this study (Section 4.5), we employ a segmented-trend, or piecewise, regression model for *dfr* [68,69,90,92,94]. The model includes linear trends for four trend segments associated with the baseline, demonstration, and two follow-up subperiods of the social experiment. Also included are possible level shifts (intercept changes) between segments. Segmented-trend regression models were employed in six previous peer-reviewed studies of this social experiment [13,44,63,64,65,70].

The regression model for the current study differs from that used in the previous study of *dfr* that examined a shorter sample of monthly data (2002–2010) [13]. The current study adds two trend segments to the previous model in order to model five years of monthly follow-up data (2012–2016) not available when the earlier study was completed. These two subperiod trend segments were included in order to better model the nonlinear behavior of *dfr*, which displays a marked increase in trend during 2015–2016 (*phase4*) relative to that during 2012–2014 (*phase3*). The *phase4* trend was an empirically based addition to the regression equation that was included in order to improve diagnostic tests of regression residuals for the full model.

The segmented-trend regression model is given by the following expression:

*Y_T_* = β_0_ + β_1_*Y_T−_*_1_ + β_2_
*T* + β_3_
*I*_1*T*_ + β_4_
*I*_1*T*_ (*T* − 60) + β_5_
*I*_2*T*_ + β*_6_ I*_2*T*_ (*T* − 120) + β_7_
*I*_3*T*_ + β_8_
*I*_3*T*_ (*T* − 156) + seasonal component + ε*_T_*, *T* = 0, 1, 2,…, 179 (1)

In Equation (1), *Y_T_* is the monthly drug-related fatality rate *dfr* in month *T*. β_0_ is the regression constant term. *Y_T_*_−1_ is the value of *dfr* in the previous month (lagged dependent variable) with regression coefficient β_1_. *T* is a monthly time counter. β_2_ is the baseline (*phase1*) time trend slope. β_3_ is the coefficient for the level-shift indicator variable *I*_1*T*_, which is a binary indicator variable equal to zero prior to January 2007 and equal to 1 thereafter. The coefficient β_4_ for the interaction term *I*_1*T*_ (*T* − 60) gives the change in trend slope from *phase1* to *phase2*. The interaction defines a monthly time counter that takes the values 1, 2, 3,…, 119 starting February 2007 and is zero prior to that month. Because the time counter starts at *T* = 0, the number 60 in the interaction term represents the 61st month of the study, January 2007.

Similarly, β_6_ quantifies the change in slope from the *phase2* to *phase3* trend that begins in January 2012. β_5_ is the regression coefficient for *I*_2*T*_ which is a binary indicator variable that equals zero prior to January 2012 and equals 1 thereafter. The time counter for the *phase3* trend segment is given by the interaction term *I*_2*T*_ (*T* − 120) = 1, 2, 3,…, 59 beginning January 2012 and equals zero before that month.

β_7_ is the coefficient for the binary level-shift indicator *I*_3*T*_, which equals 1 beginning January 2015 and is zero otherwise. β_8_ gives the change in slope from the *phase3* to *phase4* trend. The associated interaction term defines a monthly time counter for this segment, where the interaction = 1, 2, 3,…, 23 starting January 2015 and is zero prior to that month. 

The interaction variables for estimation of Equation (1) were generated using the *mkspline* command of Stata 16.1 software with “change-in-slope” coding [92,95]. This coding implies that the slope for each trend segment after *phase1* is given by the slope for the previous trend plus the change in slope for the current segment [92]. Thus, because β_2_ is the *phase1* slope, the *phase2* slope is β_2_ + β_4_, the *phase3* trend slope is β_2_ + β_4_ + β_6_, and the *phase4* trend slope is β_2_ + β_4_ + β_6_ + β_8_. The change in trend slope for *phase4* relative to the *phase2* demonstration trend is β_6_ + β_8_.

In Equation (1), the coefficients of the centered seasonal component describe the monthly seasonal variation in *dfr* that is apparent in Figure 2, thus providing seasonally adjusted estimates for the other regression coefficients. For example, as shown in Table 1, *dfr* tends to be above average during January and March and below average in September and November.

For a centered seasonal component, the seasonal coefficients for each year sum to zero. The centered seasonal indicator variables were constructed by first creating standard binary (0/1) seasonal indicators for each month. Then, we subtracted 1/*m* from each binary indicator, where *m* = 12 is the number of seasonal periods per year [96]. The centered monthly seasonal effect for each year is given by the regression coefficient *S_k_* (not shown in Equation (1)) where *k* is the month number. For each different month *k*, the value of the seasonal coefficient *S_k_* shifts the intercept in Equation (1) up or down. In estimating Equation (1), the seasonal indicator for December was omitted to avoid exact linear dependence.

Finally, the regression error term $\varepsilon_{t}$ is an independent and identically distributed, serially uncorrelated, normal “white noise” process with mean zero and variance $\sigma^{2}$.

### 4.8. Model Estimation

Equation (1) was estimated using ordinary least squares (OLS) with robust standard errors (SEs) and *t*-ratios that are corrected for possible violation of OLS statistical assumptions. The robust SEs are corrected for the possible presence of nonconstant variance of the regression error term conditional on the explanatory variables (heteroskedasticity) that can invalidate statistical tests. The “heteroskedasticity-and-autocorrelation-consistent” (HAC) SEs and *t*-ratios reported in Table 1 also correct for serial correlation of regression residuals. Positive serial correlation of the regression errors can downwardly bias SEs for the estimated regression coefficients, thus unduly inflating their *t*-statistics. HAC SEs remain valid (consistent) in the case of heteroskedasticity and/or autocorrelation of possibly unknown form [97].

The robust *t*-ratios for the regression coefficients shown in Table 1 are based on HAC SEs calculated using the Newey-West procedure with Bartlett kernel and bandwidth of four autocorrelation lags [97]. The bandwidth specifies the maximum autocorrelation lag *q* for the adjustment of SEs. It was selected automatically by PcGive 15 software as the integer part of *q* = 4(*N*/100)^2/9^, where *N* is the number of observations [98]. Sensitivity analysis indicated that the conclusions regarding Hypotheses 1 and 2 of the current study are not sensitive to the selected bandwidth (see Section 5.5).

## 5. Results

### 5.1. Estimated Regression Model

Table 1 and Table 2 provide details of the regression results for the estimate of Equation (1). The principal statistical software packages used in the analysis were the PcGive 15 module of Oxmetrics 8.1 [98] and Stata 16.1 [95]. Time Series Modelling 4.52 software [99] was also used for supplemental calculations.

**Figure 3:**
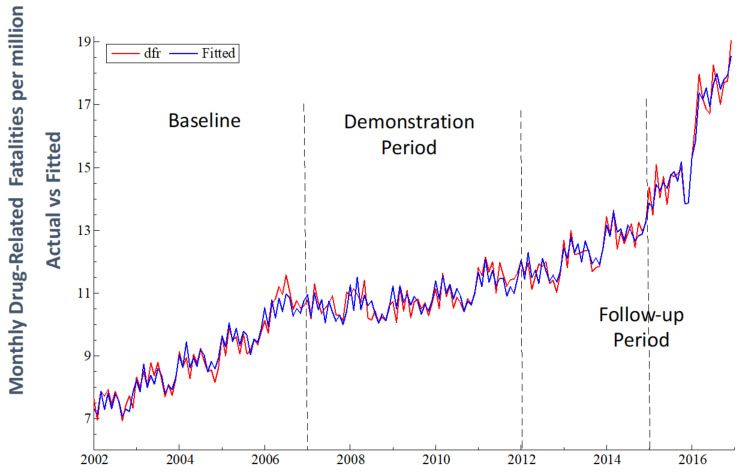
Actual monthly drug-related fatality rate (red) versus regression predicted value (blue) 2002–2016. The squared correlation between the actual and predicted *dfr* is *R*^2^ = 0.985.

The estimated regression model tracks the data well, as shown in Figure 3 and by the R-squared and adjusted R-squared in Table 2. The estimate of β_1_ indicates a significant effect of the previous month’s value of *dfr* on that for the current month. The seasonal variation of *dfr* suggested in Figure 2 and Figure 3 was confirmed by the joint significance of the seasonal regression coefficients: (*F*(11, 159) = 22.92, *p* < 0.0001).

For the demonstration phase of the study, Table 1 reports a highly significant, negative estimate for the change in trend from baseline to the *phase2* trend (β_4_). The January 2007 level shift in *dfr* (β_3_) is also significant with negative sign. These two coefficient estimates imply a reduction in the regression predicted value of *dfr* relative to the baseline trend and thus are consistent with Hypothesis 1. Likewise, the significant positive estimate for the change in trend from *phase2* to *phase3* is consistent with Hypothesis 2, as is the positive change in *phase4* trend (see Section 5.4 for formal tests of Hypotheses 1 and 2). Because there are no intercept changes for the *phase3* and *phase4* trend segments, the evaluation of Hypothesis 2 for each follow-up segment is based only on changes in trend slope.

In Table 1, the estimated coefficients for changes in trend (β_4_, β_6_, and β_8_) quantify the initial, short-run changes in *dfr* trend slopes associated with *phase2*, *phase3*, and *phase4* of the social experiment, respectively. β_3_ is an estimate of the short-run (SR) *phase2* level change. Due to the significant lagged dependent variable, Equation (1) is a dynamic regression model (stochastic difference equation) that has both SR coefficients and, if certain stability conditions are satisfied, corresponding long-run (LR) coefficient estimates. As discussed in Section 5.3, LR impacts of the MIU group on *dfr* are derived from the coefficients of the LR equilibrium (or “steady state”) solution of Equation (1). Formal tests of Hypotheses 1 and 2 are presented in Section 5.4 after operationalizing these hypotheses in terms of the estimated LR coefficient values.

The estimate of the *phase3* level shift (β_5_) in Equation (1) was small and did not approach significance (*p* = 0.26). It was dropped from the model reported in Table 1 in order to improve (lower) the Akaike information criterion (AICc) for evaluation of model specification [100]. Dropping β_5_ also improved the Bayesian information criterion (BIC) [100,101]. Both criteria are designed to balance the competing objectives of model parsimony and improved model fit. The AICc version of AIC is recommended for use in both large and small data samples [100].

Dropping the nonsignificant (*p* = 0.42) *phase4* level-shift component of the model (β_7_) also improved both the AICc and BIC, as did adding three *phase4* binary (0/1) outlier indicator variables to Equation (1) (November 2015, December 2015, and January 2016). The estimated model in Table 1 globally minimized both the AICc (118.787) and BIC (182.586) relative to the model given by simply adding these three outlier components to the full equation given in Equation (1) (AICc = 121.772, BIC = 190.661) as well as relative to other alternative models with various combinations of level shifts and outliers.

**Table 2:** Regression diagnostics for *dfr* regression results reported in Table 1.

LM test for no serial correlation ^1^: Lags 1–2: *F*(2, 157) = 2.925 (*p* = 0.057) Lags 1–7: *F*(7, 152) = 1.409 (*p* = 0.205)	LM test for no heteroscedasticity ^2^: *F*(22, 154) = 1.423 (*p* = 0.112)
LM test for normality ^3^: χ^2^ = 2.776 (*p* = 0.250)	LM test for no ARCH ^4^: Lags 1–7: *F*(7, 166) = 1.286 (*p* = 0.260)
HML test for stationarity ^5^: *z* = −0.942 (*p* = 0.70)	KPSS test for stationarity ^6^: test statistic = 0.0518 (*p* ≤ 0.88)

Note: Sample is January 2002–December 2016, *N* = 180. LM = Lagrange multiplier. 1. Breusch-Godfrey test for no autocorrelation of OLS residuals. 2. White’s test for no heteroskedasticity of OLS residuals. 3. Doornik-Hansen test for normality of OLS residuals. 4. Engle’s test for autoregressive conditional heteroskedasticity (ARCH). 5. Standard normal *N*(0, 1) test statistic under null hypothesis of stationarity of OLS residuals [102]. 6. KPSS test under null hypothesis of stationarity of OLS residuals.

### 5.2. Diagnostic Tests

Table 2 reports the results of a battery of diagnostic tests to assess whether key assumptions of the statistical analysis are satisfied.

Significant autocorrelation of regression errors is a key threat to statistical conclusion validity in TS regression modeling. The null hypothesis of no serial correlation of the OLS residuals at lags 1–7 (see Table 2) was not rejected as well as at lags 1–12 (*F*(12, 147) = 0.833, *p* = 0.616) (not shown in table) [103]. The null hypothesis of no serial correlation, however, was not rejected at lag 1 (*F*(1, 158) = 0.267, *p* = 0.606) (not shown), but was rejected at lags 1–2 (see Table 2). However, the lag 2 autocorrelation coefficient was small (0.11).

Conditional on the presence of autocorrelation at lags 1–2, no significant autocorrelation at lags higher than 2 (up to lag 12) was detected by the Cumby-Huizinga (C-H) test for autocorrelation [104]: (χ^2^(1) = 0.697, *p* = 0.404). Thus, the C-H test indicates that the HAC adjustment of SEs for the presence of autocorrelated errors up to lag 4 (Newey-West bandwidth *q* = 4) by PcGive 15 was adequate to correct SEs and *t*-statistics for the regression coefficients. The C-H test was calculated using Stata 16 add-in module *actest* [105].

Adding lag 2 of the dependent variable *dfr* to the model that already included lag 1 *dfr* worsened (increased) both the AICc (121.199) and BIC (187.960) relative to the model with only lag 1 of *dfr* (AICc = 112.341, BIC = 182.586). The lag 2 coefficient for *dfr* did not approach significance (*p* = 0.81). Thus, the model reported in Table 1 with only lag 1 of *dfr* was retained for the purpose of testing the research hypotheses.

Stationarity of the regression errors is necessary for valid statistical inferences in TS regression [106,107]. A TS is said to be covariance stationary (or weakly stationary) if its mean, variance, and autocorrelations are invariant with respect to time origin [107]. Note that the relevant condition for valid inference is covariance stationarity of the dependent variable conditional on the explanatory variables—that is, stationary regression errors, not stationarity of the dependent variable itself [107].

A test for stationarity that is appropriate for regression residuals—the HML test [99,102]—does not reject the null hypothesis of covariance stationarity of the regression errors (see Table 2). The HML test supports the conclusion that *dfr* is “segmented-trend stationary,” displaying weakly stationary fluctuations around segmented trends. Thus, the regression findings in Table 1 cannot be attributed to “spurious regression” or “nonsense regression” due to nonstationarity [108].

The HML test statistic is distributed as standard Normal *N*(0,1) in large samples under the null hypothesis of stationarity [102]. The HML test was calculated using Time Series Modelling (TSM) 4.52 software [99] with the default settings (*c* = 1.0, *L* = 0.66).

The null hypothesis of stationarity of the *dfr* regression errors was also not rejected by the KPSS test, as also calculated in TSM 4.52 [99,109].

Table 2 shows that the results of all other diagnostic tests for model adequacy are satisfactory. White’s general test [110] fails to reject the null hypothesis of no heteroskedasticity of regression errors. The null hypothesis of no autoregressive conditional heteroscedasticity (ARCH) of residuals also was not rejected [111]. Finally, the null hypothesis that the regression errors are drawn from a normal distribution was not rejected by the test of Doornik and Hansen [112]. In sum, after HAC correction of SEs for modest autocorrelation of residuals at lag 2, the diagnostic tests in Table 2 are consistent with statistical conclusion validity for inferences based on the estimates in Table 1.

### 5.3. Long-Run Estimates of Regression Coefficients

As discussed in Section 5.4, the full estimated LR impacts of the MIU group on *dfr* are derived from the estimated LR values (or “LR multiplier” estimates) of the SR coefficients in Table 1. The significant estimate of β_1_ for the lagged dependent variable indicates the presence of first-order autoregressive dynamics of *dfr*. If (1 − β_1_) ≠ 0 and β_1_ is less than 1.0 in absolute value, then these dynamics imply a gradual, exponential adjustment of each coefficient from its short-run value to its equilibrium (LR) value.

A formal hypothesis test (the “PcGive unit-root *t*-test”) rejects the hypothesis (1 − β_1_) = 0 (*p* < 0.001) in favor of the alternative that |β_1_| < 1.0 [98,106]. This indicates that the estimated model in Equation (1) is dynamically stable. Thus, each of the LR changes in trend slope will converge to their mathematically expected equilibrium (or “steady-state”) values.

Because the stability condition for β_1_ is satisfied, the LR equilibrium value for the change in *phase2* trend is given by *b*_4_ = β_4_**/**(1 − β_1_) [98]. Likewise, the corresponding LR multiplier values (as well as other coefficients in Table 1) are obtained by multiplying each SR coefficient value in Table 1 by 1/(1 − β_1_).

Note that in this context the term “long run” refers to attainment of the mathematically expected (conditional mean) equilibrium value and does not necessarily imply a long period of time [113]. The multiplicative factor 1/(1 − β_1_) implies an exponential decay over time in the absolute value of the monthly impact on *dfr* as SR coefficients approach their LR value. In the current study, LR regression coefficients will be larger (in absolute value) than the immediate impacts shown in Table 1 by a factor of 1/(1 − β_1_) = 1.633. After the onset of the social experiment, approximately 61.2%, 85.0%, 95.2%, and 97.7% of the cumulative increase in the absolute value of the SR coefficients will be completed within one, two, three, and four months, respectively.

### 5.4. Tests of Research Hypotheses 

In this section, Hypotheses 1 and 2 (given in Section 4.5) are operationalized in terms of the estimated LR model coefficients in Table 3. Formal tests of the two research hypotheses are then presented based on the LR coefficients. Table 3 shows the LR multiplier estimates for the key SR coefficients in Table 1, their SEs, and test statistics. The LR seasonal coefficients are omitted to conserve space.

The tests of Hypotheses 1 and 2 are based on the treatment effects for each trend segment. These TEs are linear combinations of the estimated LR multiplier coefficients in Table 3. The SE*s* for the LR coefficients were calculated by PcGive 15 software from the SEs for the corresponding SR coefficients in Table 1 using nonlinear numerical differentiation [98,115]. Significance tests for LR multipliers using critical values from the standard normal distribution have been shown to have *p*-values close to their nominal level as well as good power [114].

As discussed in Section 4.5, Hypothesis 1 predicts a total cumulative reduction in the regression predicted value, or fitted value, of *dfr* during *phase2*. The total *dfr* reduction by the end of the demonstration period is assessed relative to the value of *dfr* that would be predicted by the (counterfactual) continuation of the baseline trend through the end of *phase2*. As noted previously, this is a standard measure of treatment effect in ITS analysis of quasi-experiments. Thus, equivalently, Hypothesis 1 can be concisely stated as predicting that the treatment effect for the demonstration period should be negative, or TE_1_ < 0.

For evaluating Hypothesis 1, the TE for the demonstration phase (denoted as TE_1_) is operationalized in terms of a linear combination of LR regression coefficients for the *phase2* trend segment [68,69,90,92]. TE_1_ is obtained by subtracting the predicted value for *dfr* based on the counterfactual LR equation (as evaluated at the end of *phase2*, December 2011) from the corresponding predicted value for *dfr* based on the LR coefficients of the segmented-trend regression equation for the observed data (see file S2_Appendix_B in the online Supplemental Material posted at the permanent online Open Science Framework repository https://osf.io/vbkfc/). After substituting the LR coefficient values *b*_3_ and *b*_4_ for their SR equivalents β_3_ and β_4_, the subtraction of counterfactual from observed regression predicted values yields the following expression for TE_1_:

TE_1_ = *b*_3_*I*_1*T*_ + *b*_4_*I*_1*T*_ (*T* − 60).

TE_1_ is calculated by evaluating this expression at *T* = 119 (December 2011) and setting *I*_1*T*_ = 1:

TE_1_ = *b*_3_ + *b*_4_ (119 − 60) = *b*_3_ + *b*_4_ (59).

The statistical significance of treatment effects was evaluated using Stata’s *nlcom* command for evaluation of nonlinear restrictions on the regression coefficients. The resulting HAC SEs were calculated via the delta method. Consistent with Hypothesis 1, the value of TE_1_ is negative and statistically significant, TE_1_ = −3.167*, z* = −5.69, *p* < 1 × 10^−9^ with 95% CI [−4.257, −2.076].

One measure of practical significance is the percent reduction in *dfr* implied by TE_1_ relative to the mean baseline value of *dfr* (8.930 fatalities per million population). This raw effect size indicates a reduction of 35.5% (or 7.1% annually, on average) during the five years of *phase2* compared to the five-year mean monthly baseline rate. An additional measure of practical significance is the standardized effect size for the estimate of the LR change in trend slope during *phase2*: *f* = −0.703, a large effect.

The practical significance of the result for TE_1_ is also indicated by TE_1_ measured in units of the baseline SD of *dfr* (SD = 1.150): TE_1_ = −2.754 SDs (or −0.551 SDs per year), a sizeable effect. Thus, in sum, TE_1_ is negative, consistent with Hypothesis 1, and both practically and statistically significant.

Similarly, the TE for evaluating Hypothesis 2 is TE_2_. TE_2_ quantifies the difference between the LR predicted value of *dfr* at the end of *phase3* (December 2014) and the corresponding counterfactual predicted value of *dfr*. Hypothesis 2 predicts that TE_2_ should have positive sign. The counterfactual predicted value is based on the extrapolation of the demonstration-period trend through *phase3*. TE_2_ is given by evaluating the following expression at *T* = 155:

*b*_5_*I*_2*T*_ + *b_6_ I*_2*T*_ (*T* − 120)

After substituting *T* = 155, *I*_2*T*_ = 1, and setting *b*_5_ equal to zero, TE_2_ is given by the following:

TE_2_ = *b*_6_ (155 − 120) = *b*_6_ (35).

Consistent with Hypothesis 2, the value of TE_2_ is positive and statistically significant: TE_2_ = 1.286*, z* = 4.49, *p* < 1 × 10^−7^ with 95% CI [0.746, 1.900]. Relative to mean *dfr* during the demonstration period (10.853), TE_2_ represents an 11.8% increase in *dfr* (3.9% annually). In units of the baseline SD of *dfr*, TE_2_ = 1.182 SDs or 0.372 SDs per year. Additionally, the standardized effect size for the LR estimate of the increase in trend for *phase3* relative to the *phase2* trend is *f* = 0.393, a medium effect. Thus, TE_2_ is both statistically and practically significant, supporting Hypothesis 2.

Note that when the estimated level shift (intercept) component for any segment of the segmented-trend regression is not present in the regression model, (such as for the *phase3* and *phase4* trend segments here), the TE for that segment is then a function only of its trend-change coefficient multiplied by a constant. In this case, tests of the relevant research hypothesis reduce to a test of the significance of the *dfr* trend-change coefficient for that segment.

The TE for *phase4*, the second follow-up subperiod of the study, is given by TE_3_ = (*b*_6_ + *b*_8_)(23). Hypothesis 2 also predicts that TE_3_, like TE_2_, should have positive sign. Consistent with Hypothesis 2, the value of TE_3_ is positive and statistically significant: TE_3_ = 5.141*, z* = 20.10, *p* < 1 × 10^−46^ with 95% CI [4.639, 5.642]. Relative to the *dfr* mean during the demonstration period, TE_3_ is a 47.4% increase in *dfr* (23.7% annually). In units of the baseline SD of *dfr*, TE_3_ = 4.470 SDs or 2.235 SDs per year. Additionally, the standardized effect size for the LR estimate of the increase in trend for *phase4* relative to the *phase2* trend is also large: *f* = 1.594. Thus, in sum, both TE_3_ and TE_2_ are practically and statistically significant, further supporting Hypothesis 2.

**Figure 4:**
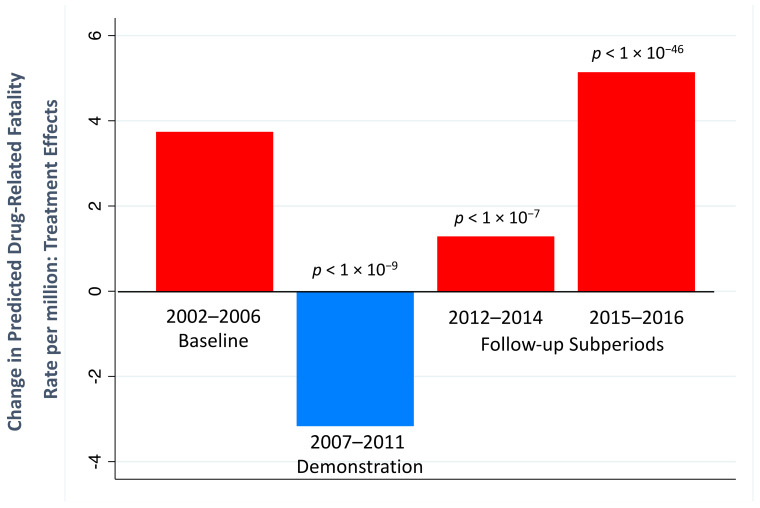
ITS treatment effects for demonstration and follow-up periods. The left red bar shows the change in regression predicted value for the monthly drug-related fatality rate (*dfr*) during the baseline 2002–2006. The blue bar shows the significant ITS treatment effect (TE_1_) for the demonstration period. TE_1_ < 0 supports Hypothesis 1, indicating a total reduction in the regression predicted value for *dfr* relative to the baseline trend. The first red bar on the right shows the significant TE_2_ for the 2012–2014 follow-up subperiod. TE_2_ > 0 supports Hypothesis 2, indicating a total 2012–2014 increase in the predicted value of *dfr* relative to the demonstration-period trend. Likewise, the significant TE_3_ > 0 (right red bar) supports Hypothesis 2, indicating a 2015–2016 total increase in the predicted value of *dfr* relative to the demonstration-period trend.

Figure 4 displays a bar graph of these results. The first bar on the left shows the increase in predicted value of *dfr* during the baseline period. The three bars on the right show the LR treatment effects associated with the *phase2* demonstration period (TE_1_) as well as the *phase3* (TE_2_) and *phase4* (TE_3_) follow-up subperiods of the 15-year quasi-experiment.

**Figure 5:**
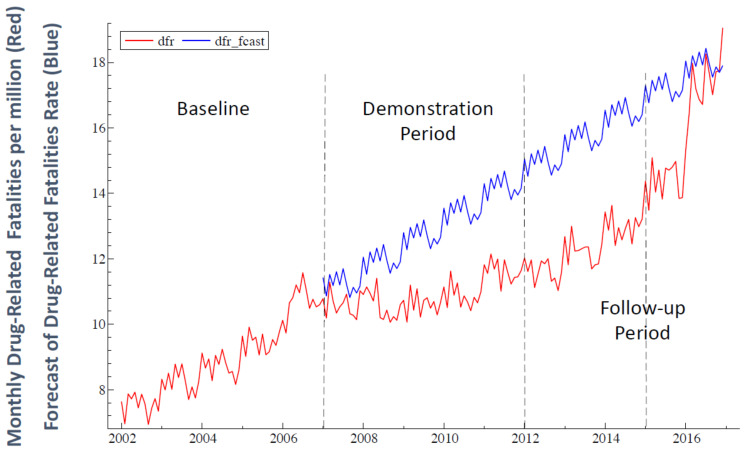
Time series plot of observed *dfr* (red) and the counterfactual forecast of future values of *dfr* (blue) for the demonstration and follow-up periods (January 2007–December 2016). The ex-ante, dynamic, one-step-ahead forecasts [98] are based on the baseline data only.

The practical significance of these empirical results is further illustrated in Figure 5, which displays a time series plot of observed *dfr* as well as the counterfactual forecast of future values of *dfr* for the demonstration and follow-up periods (January 2007–December 2016). The ex-ante, dynamic, one-step-ahead forecasts [98] are based on the baseline data only, and the regression model for generating forecasts includes the baseline trend, intercept, and seasonal components. For calculating the one-step-ahead forecast of *dfr* for the next month, the model uses the value of the lagged dependent variable from the previous month’s *dfr* forecast rather than the observed *dfr* value (for further details see file S3_Appendix_C in the online Supplemental Files posted at the permanent online Open Science Framework repository https://osf.io/vbkfc/).

Figure 5 shows that the counterfactual forecast of *dfr* lies consistently above observed *dfr* for the entire ten-year span of the combined demonstration and follow-up periods until the final two months of the sample in 2016. The significant decrease in *dfr* trend (and intercept) relative to baseline trend during the five-year demonstration period was associated with an estimated reduction of 34,194 (6839 per year) drug-related fatalities. During the follow-up phase of the study (2012–2016), when the *dfr* trend began to increase again but *dfr* remained below the projected baseline trend, an additional estimated 52,115 drug-related lives may have been saved. Thus, a total estimated reduction of 86,309 drug-related fatalities (8631 per year) may have been associated with the combined demonstration and follow-up periods of the study 2007–2016 (see Figure 5).

### 5.5. Sensitivity Analyses

For the purpose of sensitivity analysis, the regression equation that generated the OLS estimates reported in Table 1 was re-estimated by varying key features of the analysis. First, we varied the maximum number of residual autocorrelations (bandwidth *q* = 4) used in calculating the HAC SEs to 7 and 12. Second, we added a level shift at January 2012 to the regression equation that generated the results reported in Table 1. Third, the regression equation reported in Table 1 was re-estimated after omitting the three outlier indicator variables during *phase4* of the study (November 2015, December 2015, and January 2016). In each case, continued strong support for Hypotheses 1 and 2 was found (all *p*-values ≤ 0.0005). Together with the results of diagnostic tests reported in Section 5.2, this further supports statistical conclusion validity. For details see file S4_Appendix_D in the online Supplemental Files posted at the permanent online Open Science Framework repository (https://osf.io/vbkfc/).

The following section summarizes the empirical findings for Hypotheses 1 and 2 and considers alternative possible explanations for the results.

## 6. Discussion

### 6.1. Summary of Empirical Results

The findings presented in Section 5.4 offer strong empirical support for the two research hypotheses of this study. Segmented-trend regression was used to analyze monthly CDC data on U.S. drug-related fatality rates (*dfr*) 2002–2016 using an ABA, or “baseline-reversal,” interrupted time series quasi-experimental design for the prospective social experiment. The study investigated monthly *dfr* trends during the five-year baseline period 2002–2006, a five-year “demonstration period” 2007–2011, and a five-year follow-up period 2012–2016. Empirical support was found for both research hypotheses. Hypothesis 1 predicted that practice of the TM and TM-Sidhi program by a group of theoretically predicted size (√1% of the U.S. population) would be associated with a reduction in the regression predicted (or fitted) value of *dfr* relative to the baseline trend during the demonstration period. As noted in Section 4.2, this total reduction in the outcome variable relative to counterfactual continuation of the baseline trend through the demonstration period is a standard measure of treatment effect in ITS analysis of quasi-experiments [68,69]. The negative sign of this treatment effect for *dfr* (TE_1_) indicates that the predicted value of *dfr* at the end of the five-year demonstration period was less than the predicted value of *dfr* based on continuation of the baseline trend (*p* < 1 × 10^−9^), thus supporting Hypothesis 1. This finding reflects the combined effect of a significant reduction in both *dfr* level (intercept) and *dfr* trend slope relative to the baseline trend.

The estimated total percent decrease in monthly *dfr* during the demonstration period was 35.5%, calculated relative to the baseline mean, indicating that the *dfr* decline was substantively as well as statistically significant. Cohen’s [62] standardized effect size for the treatment effect was also large (*f =* −0.703). Substantive significance of the decrease in *dfr* was also indicated by the estimated 34,194 (6839 per year) drug-related fatalities that may possibly have been averted as a result of the decline in *dfr* during the demonstration phase.

Support for Hypothesis 2 was also found. It predicted that during the follow-up period of the study, a decrease in the size of the TM-Sidhi group below the theoretically required size (√1% of the U.S. population) would be associated with an increase in the regression predicted value of *dfr* relative to the demonstration-period trend. As hypothesized, the decline in the regression predicted value of *dfr* during the demonstration period was followed by increases in predicted *dfr* during the first follow-up subperiod (2012–2014) (*p* < 1 × 10^−7^) as well as the second follow-up subperiod (2015–2016) (*p* < 1 × 10^−46^). The positive sign of the treatment effect for both subperiods (TE_2_ and TE_3_) indicates that the regression predicted (or fitted) value of *dfr* at the end of each subperiod was higher than the counterfactual predicted value based on projection of the demonstration-period trend. Equivalently, because there was no significant change in *dfr* level (intercept) during either subperiod, the positive treatment effect in each case reflects a significant increase in monthly *dfr* trend slope relative to the five-year demonstration-period trend slope.

These changes in *dfr* during the two subperiods of the follow-up period were practically as well as statistically significant. During 2012–2014, the total increase in predicted *dfr* was 11.8% relative to the demonstration-period mean while the corresponding increase for 2015–2016 was 47.4%. Cohen’s standardized effect size indicates a medium effect (*f* = 0.393) for 2012–2014 and a large effect for 2015–2016 (*f* = 1.594). Practical significance was also indicated by the estimated 52,115 drug-related fatalities that may possibly have been averted during the follow-up phase of the study (see Figure 5). Thus, a total estimated reduction 86,309 fatalities may have been associated with the combined demonstration and follow-up periods 2007–2016.

As discussed in Section 5.2 and Section 5.5, diagnostic tests and sensitivity analyses support statistical conclusion validity. The following section considers other possible alternative explanations for the results reported in Section 5 that may constitute threats to internal validity.

### 6.2. Alternative Possible Explanations for the Empirical Results

The quasi-experimental treatment effects for evaluating Hypotheses 1 and 2 are both in the hypothesized but opposite directions, characteristic of a baseline-reversal, quasi-experimental effect in an ABA design. The treatment effects are highly statistically significant and substantively important in each case.

Since rejection of the null hypothesis does not automatically mean acceptance of the alternative hypothesis, it is necessary to investigate alternative explanations. The design of the study successfully eliminated many alternatives. For example, the use of robust HAC SEs for the estimated coefficients of the segmented-trend regression model controlled for effects of any serial correlation and heteroskedasticity in the regression residuals. At the same time, the use of seasonal parameters in the regression model ensured that monthly seasonal fluctuations were not responsible for the measured results. The design of the experiment also took into account the time series behavior of *dfr* before and after the demonstration period in order to most reliably assess changes during the demonstration period. Thus, the results of the interrupted time series analysis also do not appear to be plausibly explained by pre-existing trends.

Additionally, a battery of diagnostic tests indicated that the results of the analysis cannot be attributed to “spurious regression” due to nonstationarity or to questionable statistical conclusion validity due to violation of other key OLS regression assumptions. Also, sensitivity analyses indicated that the statistically significant estimates for treatment effects, and the resulting empirical support they provide for research Hypotheses 1 and 2, are robust to changes in specification of the regression model, including the bandwidth used in calculating HAC SEs, specification of level shifts, and outlier adjustment (see file S4_Appendix_D in the online Supplementary Materials posted at the permanent online Open Science Framework repository https://osf.io/vbkfc/).

Within the time frame studied, a candidate for an alternative cause of change in drug-related fatality rates is the economic disruption of 2008 and associated 2008–2009 recession, the most severe at that time since the Great Depression. One may inquire whether there could be a link between these issues and the observed decline in *dfr* trend during the demonstration period 2007–2011. However, the financial problems became acute mid-to-late 2008, whereas the decline in *dfr* trend during the demonstration period began in January of 2007. Thus, this alternative explanation is implausible.

Another possibility is that physicians became more aware of the dangers of opioids and began reducing opioid prescriptions during the 2007–2011 demonstration period. However, the peak for doctor prescription of opioids was in 2011, and only from 2012 through 2020 did physicians reduce their rate of prescribing opioids. This reduction did not result in a decline in drug-related fatalities [4] and *dfr* returned to its rapidly rising trend in the follow-up period 2012–2016.

The possible impact of temperature on drug fatality rates should also be considered. The seasonal component of the model statistically controls for any variation in *dfr* due to monthly seasonal factors, producing seasonally adjusted regression coefficients. However, possible impacts of rising trends in temperature associated with global warming also require consideration because such trends may not have been fully accounted for by the seasonal component of the regression model. Consideration of this factor is important because high ambient temperatures have been shown to be associated with a significant increase in mortality from cocaine overdose [116]. Average temperature levels differed during the demonstration and follow-up periods of the current study. During the demonstration period, average monthly temperatures were falling for the contiguous U.S. states. NOAA statistics show a small decline of 0.59 °F (3.8% of baseline temperature SD) relative to the five baseline years [117]. In the follow-up period, average monthly temperatures increased by approximately 1 °F (6.7% of baseline SD) relative to baseline.

To control for the possible effect on *dfr* of differences in average temperature, the (centered) average monthly temperature (*ctemp*) was added to the regression model reported in Table 1. The added temperature variable was not significant in the expanded model (*t*(158) = 0.00, *p* = 0.998), and all other SR and LR coefficients were virtually unchanged from those reported in Table 1 and Table 3. Thus, after explicitly controlling for temperature, the estimated treatment effects for testing Hypotheses 1 and 2 were unchanged, and strong empirical support for both hypotheses continued to be found (both *p* < 0.0001).

In sum, it is difficult to conceive of plausible alternative explanations for both the statistically and practically significant decrease in *dfr* relative to the five-year rising baseline trend during the demonstration phase of this prospective social experiment as well as for the subsequent significant change from a declining trend to an increasing trend in the follow-up phase (baseline reversal effects). These changes in trend in opposite directions at the predicted times, as postulated in Hypotheses 1 and 2, offer strong statistical support for both research hypotheses examined in the current study.

### 6.3. Possible Mechanism of Action

Based on the results of studies such as the present one, the dynamics of action for the hypothesized effects on society of group practice of the TM and TM-Sidhi program is an appropriate question for future research. It is anticipated that this avenue of research investigating the mechanism of action of the group practice of the TM and TM-Sidhi program from the point of view of modern science will expand as a result of the present study. Quantum physicist John Hagelin and a team of other researchers at MIU are actively investigating this topic. Additionally, another prior attempt to empirically address this question found that non-meditators outside the TM-Sidhi group showed a corresponding change in a number of biochemical parameters as the size of the TM-Sidhi group at MIU varied [118].

Using dynamic regression analysis of time series observations over the experimental period (77 days), Walton et al. [118] found that an increase in the day-to-day change in the size of the TM-Sidhi group for the afternoon session was a significant predictor of the subsequent reduced overnight excretion rate of the stress hormone cortisol in a group of 6 non-meditators living and working up to 20 miles from the group. An increase in the daily change in group size also was a significant predictor of future overnight increases in both the excretion rate of the main metabolite of serotonin (5-HIAA) and the ratio of the excretion rates of 5-HIAA to cortisol. These findings are consistent with those of a prior time series quasi-experiment that examined 5-HIAA excretion in another sample of individuals outside the group. Thus, the authors concluded that these results support the hypothesis that group practice of the TM and TM-Sidhi program reduces social stress by producing a beneficial neuroendocrine effect in non-meditators outside the group.

In contrast to the search for an explanation of the mechanics of action of group TM-Sidhi practice from the point of view of physical science, Maharishi Mahesh Yogi explains the dynamics of action from the point of view of the Vedic understanding of consciousness as brought to light in Maharishi Vedic Science and Technology (see Section 2.4). From this perspective, the positive effects on society of group practice of the TM and TM-Sidhi program are produced by enlivenment of the universal field of pure consciousness in the collective consciousness of society, thus reducing social stress and positively influencing individual thought and behavior. Pure consciousness is described as a unified field of nature’s intelligence, and it is said to be the same as the unified field identified in quantum unified field theories in physics [46,48].

From the point of view of the dominant physicalist, or materialist, paradigm in modern science, however, the extensive research findings supporting the hypothesized effects of group practice of the TM and TM-Sidhi program on society raises the question of how the field of consciousness can influence matter. This paradox is resolved in the Vedic perspective of MVST—as brought to light by Maharishi and elaborated by neuroscientist Tony Nader [47,50]—by the proposition that “consciousness is all there is.” From this point of view, the difference between physical matter and consciousness is only apparent. Thus, from this perspective, the influence on society of the group practice of the TM and TM-Sidhi program is seen as a phenomenon of consciousness interacting with itself, transforming itself.

### 6.4. Possible Causal Interpretation

The theoretically predicted successive changes in trend in the opposite direction at the theoretically predicted times are consistent with—but, of course, do not necessarily imply—a causal interpretation of these quasi-experimental results. That these changes in *dfr* trends do not appear to be plausibly explained by other variables known to influence *dfr* could also be argued to support a possible causal interpretation.

Further support for a possible causal interpretation of the current empirical results is offered by six additional characteristics of the peer-reviewed empirical literature on societal effects associated with group practice of the TM and TM-Sidhi program (reviewed in Section 2.6 and Section 3): (1) *dose effects*: larger TM-Sidhi groups are associated with empirical evidence of larger social effects; (2) *replication*: empirical support for the hypothesized societal effects of TM-Sidhi groups have been reported in 27 other peer-reviewed articles published in independent, scholarly journals; (3) *temporal precedence*: evidence of temporal precedence is provided, for example, by cross-lagged panel analysis, cross-correlation analysis in transfer function studies, or lagged impact-assessment effects that reveal a unidirectional lead-lag relationship in which changes in the size of the TM-Sidhi group (or percentage of TM meditators in a city) preceded predicted changes in social outcomes; (4) *predictions lodged in advance*: further evidence of temporal precedence is provided by studies for which outcomes of prospective studies were lodged in advance with formal independent review boards and/or the press; (5) *predictions based on theory*: causal interpretations of observed empirical associations require that the posited causal effects are based on an explicit theoretical framework, such as MVST, that generates empirically testable predictions; and (6) *beneficial changes in numerous social indicators*: observed changes in the broad range of social indicators associated with group practice of the TM and TM-Sidhi program are consistent with the theoretical prediction that these diverse observed changes in social behavior are a result of effects created on the holistic, universal level of pure consciousness, which is posited to underly and influence the more expressed values of individual and collective consciousness.

## 7. Conclusions

In conclusion, this study evaluates evidence for the effectiveness of a proposed Consciousness-Based approach intended to possibly help reduce rising U.S. trends of drug-related mortality through group practice of the TM and TM-Sidhi program. The findings of the segmented-trend ITS regression analysis summarized in Section 6.1 replicated and extended previously published results for drug fatality trends during the same quasi-experiment, where the previous findings were based on data from shorter baseline and demonstration periods [13].

The findings of the current study are consistent with those of six prior research studies on this social experiment (see Section 2.6) that also supported the hypothesis that practice of the TM and TM-Sidhi program by a group of theoretically predicted size (√1% of the U.S. population) would be associated with statistically and practically significant reductions in a diverse set of negative U.S. public health indicators relative to baseline trends [13,44,63,64,65,70]. As in the current study, the improvement in these indicators could not be plausibly explained by alternative factors. Like the current study, two of these prior studies also included follow-up data [44,70]. As in the present research, these two prior studies reported significant reductions of negative public health indicators during the demonstration phase that were followed by significant increases in these indicators relative to the demonstration-period trend when the size of the TM-Sidhi group fell below the critical √1% level.

The results of the current research are also consistent with those of 21 other prior peer-reviewed articles reviewed in Section 3 (many examining multiple outcome variables) that analyzed data other than that from the current social experiment. This additional research also provides empirical support for hypothesized improvements in a wide array of public health and other social indicators predicted to be associated with group practice of these technologies of consciousness.

Taken together, the findings of this study plus those from 27 other peer-reviewed empirical studies would seem to provide sufficient evidence to merit public and private sector support for the establishment, maintenance, and further rigorous evaluation of the hypothesized effects of a stable TM-Sidhi group in the U.S. consisting of at least the √1% of the U.S. population. Such a group is intended to supplement the hypothesized impact of public-heath initiatives to increase practice of the TM program by individuals in society. Permanent endowment of such a group is estimated to cost less than one U.S. B-2 bomber aircraft. In the absence of other effective solutions to the intractable twin epidemic of rising drug-related death and pervasive social stress, the evidence-based approach discussed in the current research would seem to merit serious consideration for timely implementation and further empirical evaluation.

## Figures and Tables

**Table 1:** OLS regression estimates for monthly drug-related fatality rate (*dfr*).

Regression Coefficient	Coeffic. Estimate	Standard Error (SE) ^1^	*t*-Ratio ^2^
Constant term (β_0_)	4.369	0.669	6.54 ^a^
Lagged dep. variable (β_1_)	0.388	0.091	4.25 ^a^
Baseline trend (β_2_)	0.038	0.005	7.18 ^a^
Level shift Jan. 2007 (β_3_)	−0.358	0.148	−2.42 ^d^
Trend shift Jan. 2007 (β_4_)	−0.027	0.005	−5.27 ^a^
Trend shift 2012 (β_6_)	0.022	0.007	3.42 ^b^
Trend shift 2015 (β_8_)	0.114	0.016	6.96 ^a^
January seasonal (*S*_1_)	0.489	0.128	3.82 ^b^
February seasonal (*S*_2_)	−0.285	0.145	−1.97 ^d^
March seasonal (*S*_3_)	0.676	0.133	5.09 ^a^
April seasonal (*S*_4_)	−0.297	0.150	−1.98 ^c^
May seasonal (*S*_5_)	0.241	0.115	2.10 ^c^
June seasonal (*S*_6_)	−0.371	0.137	−2.71 ^c^
July seasonal (*S*_7_)	0.237	0.121	1.96
August seasonal (*S*_8_)	−0.161	0.120	−1.34
Sept. seasonal (*S*_9_)	−0.579	0.097	−5.91 ^a^
Oct. seasonal (*S*_10_)	−0.157	0.098	−1.59
Nov. seasonal (*S*_11_)	−0.455	0.109	−4.19 ^a^
Outlier Nov. 2015	−1.250	0.088	−14.30 ^a^
Outlier Dec. 2015	−1.391	0.164	−8.50 ^a^
Outlier Jan. 2016	−0.577	0.206	−2.81^c^
*F*-statistic: *F*(20, 159) = 536.30 (*p* < 0.001)		Mean (SD) of *dfr* = 11.166 (2.427)	
Root MSE = 0.311		*R*^2^ = 0.985; Adjusted *R*^2^ = 0.984	
Sum of squared residuals = 15.406		Log-likelihood = −34.170	
AICc ^3^ = 118.787		BIC ^4^ = 182.586	

Note: Sample is January 2002–December 2016, *N* = 180. OLS = ordinary least squares. HAC = heteroskedasticity and autocorrelation consistent. LM = Lagrange multiplier. 1. HAC SEs (Newey-West) with Bartlett kernel and automatic bandwidth selection of 4 lags. 2. HAC *t*-ratios (*df* = 159). 3. Akaike information criterion (AICc) appropriate for both smaller and larger samples. 4. Bayesian information criterion. The *t*-ratios are those reported by PcGive 15 and may differ from the coefficient estimate divided by SE due to rounding. Two tailed *p*-values: a. *p* ≤ 0.0001, b. *p* ≤ 0.001, c. *p* ≤ 0.01, d. *p* ≤ 0.05.

**Table 3:** Estimates of long-run equilibrium changes in *dfr* trend slopes and level.

Long-Run Equilibrium Coefficient (LR Multiplier) ^1^	Coefficient Estimate	Standard Error (SE) ^2^	*t*-Ratio ^3^
Constant (*b*_0_)	7.137	0.132	54.20 ^a^
Baseline slope (*b*_2_)	0.062	0.004	16.30 ^a^
Level shift 2007 (*b*_3_)	−0.585	0.183	−3.20 ^b^
Trend shift 2007 (*b*_4_)	−0.044	0.005	−8.87 ^a^
Trend shift 2012 (*b*_6_)	0.037	0.007	4.96 ^a^
Trend shift 2015 (*b*_8_)	0.187	0.015	12.50 ^a^

Note: Sample is January 2002–December 2016, *N* = 180. 1. LR coefficient estimates from the LR equilibrium equation for *dfr* are derived from the OLS regression estimates in Table 1. 2. SEs derived from the SEs in Table 1 by nonlinear numerical differentiation. 3. The *t*-ratios reported by PcGive 15 were evaluated for statistical significance using the standard normal distribution [114]. Due to rounding, the *t*-ratios may differ from the coefficient estimate in the table divided by the table SE. Two tailed *p*-values: a. *p* ≤ 0.0001, b. *p* ≤ 0.001, c. *p* ≤ 0.01, d. *p* ≤ 0.05.

## Data Availability

Although the data analyzed in this study are public source, they are also posted at the permanent online Open Science Framework repository https://osf.io/vbkfc/, accessed on 15 January 2023.

## Supplementary Materials

The following files are available online at the permanent online Open Science Framework scholarly repository for this study (https://osf.io/vbkfc/, accessed on 15 January 2023). S1_Appendix_A (Effect size for regression coefficients); S2_Appendix_B (Derivation of test statistics for Hypotheses 1 and 2); S3_Appendix_C, (Forecast of Counterfactual Rate of Drug-Related Fatalities); S4_Appendix_D (Sensitivity analyses); and S5_Stata_data_file.

## Trademarks

Transcendental Meditation, TM, TM-Sidhi, Maharishi, Maharishi Vedic Science and Technology, MVST, Consciousness-Based, and other terms used in this paper are protected trademarks and service marks. All are used under license or with permission.
